# Supplementary material for: Content-rich biological network constructed by mining PubMed abstracts
Source: BMC Bioinformatics. 2004 Oct 8;5:147. doi: 10.1186/1471-2105-5-147 (PMC528731; doi:10.1186/1471-2105-5-147)
Supplement: Additional File 2 — The original results of the above study (non-essential files are deleted to keep the file size under the limit set by BMC bioinformatics). [file 1471-2105-5-147-S2.bz2 › chilibotAdditionalFile2/dip05/254ID7809159E1012/html/CKS1_CDK3.html]

 


 **CKS1** and **CDK3** 
  
Found 1 abstracts in PubMed, retrieved 1.  
 

 What does Google say? 
 PDF only 
| .edu only 

---

**Interactive relationship** (e.g. stimulation, inhibition, etc)

**Neutral relationship**- Despite the high degree of homology between the nine known CDKs, only CDK1, CDK2 and, to a lesser extent,  **CDK3**  are able to bind to the suc1 Cks  [ **CKS1** ]  proteins.  Ref: 12007796 Biochim Biophys Acta, 2002
